# Optimal Water Resources Regulation for the Pond Irrigation System Based on Simulation—A Case Study in Jiang-Huai Hilly Regions, China

**DOI:** 10.3390/ijerph16152717

**Published:** 2019-07-30

**Authors:** Shangming Jiang, Shaowei Ning, Xiuqing Cao, Juliang Jin, Fan Song, Xianjiang Yuan, Lei Zhang, Xiaoyan Xu, Parmeshwar Udmale

**Affiliations:** 1 Key Laboratory of Water Conservancy and Water Resources of Anhui Province, Water Resources Research Institute of Anhui Province and Huaihe River Commission, Ministry of Water Resources of the People’s Republic of China, Hefei 230088, China; 2 School of Civil Engineering, Hefei University of Technology, Hefei 230009, China; 3 Institute of Water Resources and Environmental Systems Engineering, Hefei University of Technology, Hefei 230009, China; 4 Information Center (Hydrology and Water Resources Monitoring and Forecasting Center), The Ministry of Water Resources of the People’s Republic of China, Beijing 100053, China; 5 Shenyang Academy of Environmental Sciences, Shengyang 110000, China; 6 Department of Civil and Earth Resources Engineering, Kyoto University, Kyoto 615-8540, Japan

**Keywords:** water resources regulation, simulation, water-saving irrigation method, Jiang-Huai hilly regions

## Abstract

Due to the importance and complexity of water resources regulations in the pond irrigation systems of the Jiang-Huai hilly regions, a water allocation simulation model for pond irrigation districts based on system simulation theory was developed in this study. To maximize agricultural irrigation benefits while guaranteeing rural domestic water demand, an optimal water resources regulation model for pond irrigation districts and a simulation-based optimal water resources regulation technology system for the pond irrigation system were developed. Using this system, it was determined that the suitable pond coverage rate (pond capacity per unit area) was 2.92 × 10^5^ m^3^/km^2^. Suitable water supply and operational rules for adjusting crop planting structure were also developed the water-saving irrigation method and irrigation system. To guarantee rural domestic water demand, the multi-year average total irrigation water deficit of the study area decreased by 4.66 × 10^4^ m^3^/km^2^; the average multi-year water deficit ratio decreased from 20.40% to 1.18%; the average multi-year irrigation benefit increased by 1.11 × 10^5^ RMB (16,128$)/km^2^; and the average multi-year revenue increased by 6.69%. Both the economic and social benefits were significant. The results of this study provide a theoretical basis and technological support for comprehensive pone governance in the Jiang-Huai hilly regions and promote the establishment of a water allocation scheme and irrigation system for pond irrigation districts, which have practical significance and important application value.

## 1. Introduction

In small water-storage irrigation projects, ponds are created by excavating the ground or formed in depressions, which then intercept and store local surface runoff. They play an essential role in the conservation of water for irrigation and offer a means of collecting monsoon precipitation to provide for agricultural and household needs over the entire year [1,2]. Small pond irrigation systems have long attracted the attention of those in charge of rural development and can be found around the world [3,4,5]. According to a water resources survey in China, by the end of 2011 there were 4,565,100 ponds with a total capacity of 30.317 billion m^3^. They were mainly distributed in hilly regions, accounting for about 71% of the total number of pond projects [6]. Pond irrigation has a long history in Jiang-Huai hilly regions. In 210 Already changed (A.D.), during the Jian’an reign of the Eastern Han dynasty, Liu Fu, Prefectural Governor of Yangzhou and stationed in Hefei, recruited refugees to open up wasteland, grow food grain, build dams and weirs, impound reservoirs, and irrigate farmland [7]. These official impounding reservoirs and ponds built by the people were parts of a pond irrigation project system in the hilly regions, which occupied an important place in the development history of water conservation [8]. According to the statistics, by the end of 2010 there were 479,200 ponds in Jiang-Huai hilly regions of Anhui Province with a total capacity of 2.758 billion m^3^ and an average annual total water supply of 3.861 billion m^3^ [8]. Existing ponds in the Jiang-Huai hilly regions were mostly built in the 1950s or 1960s, and were characterized by low overall project construction standards, unknown responsibility, and unclear management systems. Left unattended and unused, they have either been lost to sedimentation or transformed into farmland, which has resulted in a sharp reduction in both the number of ponds and their storage capacity [6]. This has reduced water resources supply to meet demand, seriously affecting the security of food production and development of the rural economy in pond irrigation districts. The issue of how to regulate, store, and allocate rainwater, surface water, pond storage and soil water in the pond irrigation districts therefore remains. Therefore, the development of a scientific and effective water resource utilization and management strategy to satisfy the agricultural irrigation water demand in an environmentally-friendly manner has become an urgent matter for decision makers.

The optimal allocation of water resources in irrigation districts has always been at the frontier of water conservancy science and has received extensive attention from experts all over the world with beneficial research results [9,10,11,12,13,14,15]. However, earlier studies mostly focused on large and medium-sized irrigation districts at the macroscopic scale, with very few on ponds, weirs, or other small water-storage irrigation districts [16,17]. Using system analysis and coordination theory to solve optimization algorithms for various allocation models, optimal water allocation schemes were developed [18]. However, they have rarely simulated the storage-irrigation-consumption-loss of field water and the mutual transformations at the microscopic field scale, or determined the optimal allocation of water resources in irrigation districts based on water supply and demand balance analysis. For ponds and other small water conservancy projects that are widely distributed in the field, optimal water resources regulations are essentially different from those of the multi-water user and multi-objective optimal water resources regulations of reservoirs [19,20,21]. At present, the primary and most effective way of regulating pond water resources is through crop planting proportioning, pond capacity setting (pond coverage rate), and water-saving crop irrigation methods [22,23,24,25].

In this study, a typical pond irrigation district in the Jiang-Huai hilly regions was selected as an example. Based on multi-year irrigation experiment, a field-scale water allocation simulation model according to system simulation theory was developed. An optimal water resources regulation system and a water-saving irrigation method for the pond irrigation district were also developed. These can provide the theoretical basis for comprehensive governance of ponds and technical support for the optimal management and sustainable utilization of water resources in the Jiang-Huai hilly regions.

## 2. Study Area

**Figure 1:**
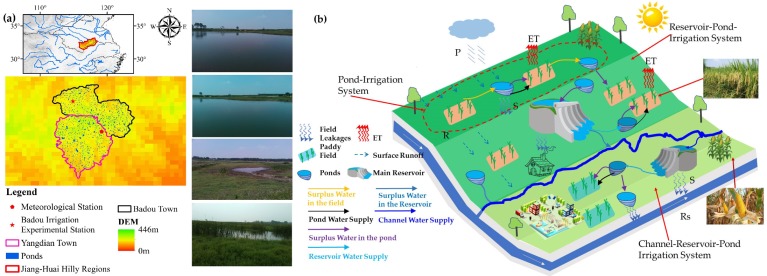
Location of the study area (**a**) and diagram of Pond-Irrigation system in the Jiang-Huai hilly region (**b**).

Yangdian Town is located in northern Feidong County, Anhui Province in China (as shown in Figure 1). It is along the ridge of the Jiang-Huai Watershed. It has a land area of 88.5 km^2^, and a farmland area of 51 km^2^. Its total population is 35,400, of which the agricultural population is 22,000, so it is a typical agricultural town. There are a total of 19 villages in the region. The ground elevation is 44–100m and is concave in shape. The terrain is fragmented and poorly regulated, and water storage capacity is low, which results in difficulties in intercepting and storing surface runoff. Ponds and small reservoirs are mainly used to store water, and there are numerous areas that are not suitable for water conservation, so the guaranteed irrigation rate is low [3,11]. According to China’s water resources survey, Yangdian Town currently has 134 ponds with a capacity more than 50,000 m^3^, and 1037 ponds with a capacity between 500–50,000 m^3^. The total pond capacity is 0.62 × 10^7^ m^3^. The pond irrigation district of Yangdian Town has an average pond capacity of 1.02 × 10^6^ m^3^/km^2^ and the irrigation area is 57.8 km^2^. Major crops include rice (medium rice or single-cropping late rice), wheat, and corn. Crop rotation is by double-cropping rotation of rice-wheat or corn-wheat. The planting proportion of autumn rice is about 0.52. Characterized by water shortages, a dry climate, and barren soil, Yangdian Town is a low-yield district with a typical pond irrigation system in the Jiang-Huai hilly regions. As shown in Figure 1b, pond irrigation system (highlighted by red-dot line) is the basic irrigation mode and also the most import component of Reservoir-Pond and Channel-Reservoir-Pond irrigation system over the Jiang-Huai hilly region.

## 3. Method

### 3.1. SCS Model-Based Rainfall Runoff Simulation for the Pond Irrigation System

Earlier studies have shown that the Soil Conservation Service (SCS) curve number (CN) model is an effective approach for solving pond irrigation hydrological problems in ungauged areas [26]. The basic rainfall-runoff relationship is [27]:

(1) $$R=\{\begin{array}{cc} \frac{{\left(P-\lambda S\right)}^{2}}{P+(1-\lambda )S} & P\geq \lambda S\\ 0 & P<\lambda S \end{array}$$

where, *P* is the precipitation (mm), *R* is the surface runoff (mm), *S* is the maximum potential retention of the watershed. In the US, small agricultural catchment experiments showed that *λ =* 0.2. However, the intra-annual distribution of rainfall in the US is relatively uniform and about 70% of rainfall enters the soil via infiltration. In China, there are significant seasonal variations in the rainfall distribution which includes concentrated heavy rainstorms. Therefore, only about 40% of rainfall enters the soil via infiltration. Because of the difference in the rainfall temporal distribution between the US and China, when applying the SCS-CN model to the Yangdian Town pond irrigation district, *λ* has been set to well below 0.2 and generally below 0.05. The specific value of *λ* for a catchment can be determined by calibrating the model with catchment hydrological data or using values from other catchments with similar hydrological conditions [26,27].

The range of *S* is extremely wide, so it is difficult to assign a value. Hence, a dimensionless parameter *CN* was introduced that ranges from 0–100. The empirical relationship is:

(2) $$S=\frac{25400}{CN}-254$$

where, *CN* is a dimensionless parameter that reflects the combined characteristics of Antecedent Moisture Conditions (*AMC*), vegetation, slope, soil type, and land utilization status of a catchment, as well as the influence of underlying surface conditions on runoff yield and concentration.

Using the 5d rainfall depth preceding the current rainfall, the SCS model classifies the *AMC* into three grades, i.e., *AMCI* for dry, *AMCII* for moderately moist, and *AMCIII* for moist. The *CN* of one moisture grade can be converted for others according to the following equations:

(3) $$CN_{1}=CN_{2}-\frac{20\times (100-CN_{2})}{100-CN_{2}+\exp[2.533-0.0636(100-CN_{2})]}$$

(4) $$CN_{3}=CN_{2}\times \exp[0.00673(100-CN_{2})]$$

**Figure 2:**
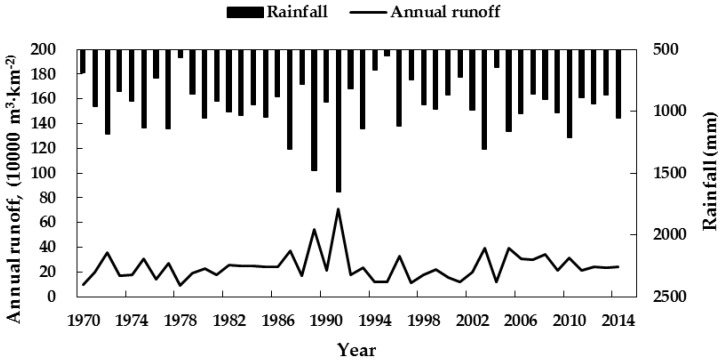
Simulated annual runoff for the Yangdian Town pond irrigation district from 1970–2014.

Jiang et al. (2013) developed a rainfall runoff simulation model for pond irrigation systems in the study area [10]. They calibrated the relevant parameters for the SCS model and simulated rainfall-runoff processes for different representative years at Yangdian Township, Badou Town, Chenji Township, and other areas. Based on previous research, this study used calibrated SCS model parameters for Yangdian Town, i.e., *λ =* 0.01, *CN*_1_ = 67.6, *CN*_2_ = 83.4, and *CN*_3_ = 93.3 (more details can be referred to the supplementary file). Due to the lack of long-term rainfall data at Yangdian Town, daily rainfall at Feidong Station (Figure 1) from 1970–2014 was used as the input to the SCS model. The SCS model with the above parameters then simulated daily runoff yields of the pond system in the study area. Further, daily runoff was calculated and used as an input for the simulation and water resources regulation of the pond irrigation system. Figure 2 shows the runoff simulation for the pond irrigation district of Yangdian Town from 1970–2014.

To meet the modeling analysis requirements for water resources regulation, frequency analysis was performed on the rainfall data series at Feidong Station from 1970–2014, resulting in average rainfall = 967.8mm, *C_v_* = 0.24, and *C_s_*/*C_v_* = 3.0. Rainfall depths for *P* = 20%, *P* = 50%, *P* = 75% and *P* = 90% were 1151.3, 939.9, 800.6 and 693.7 mm, respectively.

### 3.2. Water Allocation Simulation of the Pond Irrigation System

In this study, water allocation simulation rules for the pond irrigation system were developed based on the system simulation principle. Rice field rainfall storage depth, dry-wet alternate irrigation intermission of rice, irrigation depth, dry crop irrigation method, crop planting proportions, pond coverage rate, and other parameters were selected as variables. Using field water storage, pond storage, and other parameters as intermediate variables, a water allocation simulation model for the pond irrigation system was developed. The model can be used to simulate water allocation, transfer, and consumption processes of the pond irrigation system under different variable combinations, along with the water deficit and degree of water deficit of crops in different growth stages. The specific processes are described as follows.

#### 3.2.1. Water Resources Structure of a Pond Irrigation System

In the development of a water allocation simulation model, one of the key issues is how to generalize complicated water resources system structures by balancing and weighing the validity of hydrological data and the structures in the water resources system. In the Jiang-Huai hilly pond irrigation districts, water stored in the ponds has been used as the water source for irrigation. Individual pond projects, despite their small scale, low water storage, and small controlled irrigation area, are characterized by having a wide distribution, large number of ponds, and high total water storage capacity. In this study, the key challenge in developing the water allocation simulation model was how to generalize the 1171 ponds in the irrigation district of Yangdian Town. Taking the pond capacity per unit area as the pond coverage rate, it was both rational and practical to use this to describe the scale and layout of pond projects in a district. In the pond irrigation district of Yangdian Town, the pond coverage rate was 1.03 × 10^5^ m^3^/km^2^. In this study, the pond coverage rate was used to provide a general description of the pond number and scale. Assuming that the distribution of pond number and capacity within the study area were uniform, the model used 1 km^2^ cells to generalize ponds per km^2^ as a virtual pond, and used cell pond coverage rates as the virtual pond capacity. 

#### 3.2.2. Water Balance Equations for Basic Elements in Various Computing Units

① Field water balance equation

The field water balance equation is:

(5) $$W_{i,j}=W_{i,j-1}+P_{j}+M_{i,j}+G_{i,j}-ETc_{i,j}-S_{i,j}-X_{i,j}$$

where *W_i_*_,*j*_ and *W_i_*_,*j*-1_ are the field water storage values (mm) of the *i^th^* crop at the end and beginning of the *j^th^* period, respectively; *P_j_* is the rainfall (mm) in the *j^th^* period; *M_i_*_,*j*_ is the farmland irrigation water volume (mm) of the *i^th^* crop in the *j^th^* period; *G_i_*_,*j*_ is the direct utilization of groundwater (mm) by the *i^th^* crop in the *j^th^* period; *ETc_i_*_,*j*_ is the water requirement (mm) of the *i^th^* crop in the *j^th^* period; *S_i_*_,*j*_ is the field leakage (mm) of the *i^th^* crop in the *j^th^* period; and *X_i_*_,*j*_ is the field surplus water (mm) of the *i^th^* crop in the *j^th^* period (i.e., water overflows farmland water storage when it exceeds the storage depth threshold). As part of this study, the Daguantang irrigation district was investigated. Results showed that the soil was compact and hardened, and characterized by extremely poor permeability, low groundwater runoff, and a scattered distribution. The burial depth was between 50–60 m and the direct utilization of groundwater by crops was negligible. Therefore, in this study, *G_i_*_,*j*_ = 0.

The equation for field water storage *W_i_*_,*j*_ is:

(6) $$W_{i,j}=hc_{i,j}+\gamma H_{i}{}_{,j}\theta_{i,j}$$

where *hc_i_*_,*j*_ is the water or irrigation depth of the field (mm) of the *i^th^* crop in the *j^th^* period, (for dry crops, *hc_i_*_,*j*_ = 0); *H_i_*_,*j*_ is the calculated soil depth (mm) of the *i^th^* crop in the *j^th^* period; γ is the dry density of soil (g/cm^3^) at the calculated soil depth; *θ_i_*_,*j*_ is the soil moisture content of the *i^th^* crop in the *j^th^* period, which is proportional to the weight of dry soil.

The equation for farmland irrigation water volume *M_i_*_,*j*_ is:

(7) $$M_{i,j}=\frac{\alpha \times MQ_{i,j}}{SQ_{i,j}}\times 10^{-7}$$

where *MQ_i_*_,*j*_ is the irrigation water consumption (m^3^) of the *i^th^* crop in the *j^th^* period, i.e., the water intake from a water supply source; *α* is the effective utilization coefficient of farmland irrigation water; and *SQ_i_*_,*j*_ is the irrigation area (hm^2^) of the *i^th^* crop in the *j^th^* period.

The water requirement *ETc_i_*_,*j*_ of a crop can be calculated from the following equation [24,25]:

(8) $$ETc_{i,j}=ET_{0j}\times Kc_{i,j}$$

where *ET*_0*j*_ is the transpiration of a reference crop (mm) in the *j^th^* period; *Kc_i_*_,*j*_ is the crop coefficient of the *i^th^* crop in the *j^th^* period. Using the Penman-Monteith formula, *ET*_0_ can be calculated from the following equation [28,29]:

(9) $$ET_{0}=\frac{0.408\Delta (R_{n}-G)+\gamma \frac{900}{T+273}U_{2}(e_{a}-e_{d})}{\Delta +\gamma (1+0.34U_{2})}$$

where *ET*_0_ is the transpiration (mm/d) of a reference crop; Δ is the tangent slope of the temperature-saturation vapor pressure curve (kPa∙°C^−1^) at *T*; *R_n_* is the net radiation (MJ/m^2^∙d); *G* is the soil heat flux (MJ/m^2^∙d); *γ* is the hygrometer constant (kPa∙°C^−1^); *T* is the average air temperature (°C); *U*_2_ is the wind velocity (m/s) at a height of 2 m; *e_a_* is the saturation vapor pressure (kPa); and *e_d_* is the actual vapor pressure (kPa).

In this study, based on results from water requirement contour maps for major crops in Anhui Province [7], standard crop coefficients and modified formulas for 84 crops as recommended by the United Nations Food and Agriculture Organization (FAO), and the results of irrigation experiments at Badou irrigation experimental station over the years, the crop coefficients (*Kc*) for various crops at different growth stages were determined [28].

② Pond water balance equation

The pond water balance equation is:

(10) $$Vt_{k}{}_{,j}=Vk_{k,j-1}+P_{j}\times SSt_{k}\times 10^{-1}+Wt_{k,j}-MQt_{k,j}-St_{k,j}-Xt_{k,j}$$

where *Vt_k_*_,*j*_ and *Vt_k_*_,*j*−1_ are the water storage values (unit = 1 × 10^4^ m^3^) of the *k^th^* pond at the end and beginning of the *j^th^* period; *P_j_* is the rainfall depth (mm) in the *j^th^* period; *SSt_k_* is the water surface area (km^2^) of the *k^th^* pond; *Wt_k,j_* is the water inflow (1 × 10^4^ m^3^) of the *k^th^* pond in the *j^th^* period; *MQt_k,j_* is the agricultural irrigation water supply (1 × 10^4^ m^3^) of the *k^th^* pond in the *j^th^* period; *St_k_*_,*j*_ is the evaporation and leakage loss (1 × 10^4^ m^3^) of the *k^th^* pond in the *j^th^* period; and *Xt_k_*_,*j*_ is the surplus water (1 × 10^4^ m^3^) of the *k^th^* pond in the *j^th^* period (i.e., the water that exceeds pond storage capacity).

#### 3.2.3. Operational Rules for the Pond Irrigation System Water Allocation Simulation Model

The simulation rules of the pond irrigation system are relatively simple, as all ponds serve the sole purpose of water-storage irrigation, except for those located near the villages that also supply rural domestic water. The specific water allocation principles are as follows:

For the ponds located near the villages, their water storage is allocated under the premise of guaranteeing rural domestic water supply;

For the ponds used only for water-storage irrigation, their water storage allocation is entirely based on meeting the requirements of the agricultural irrigation under all circumstances.

**Figure 3:**
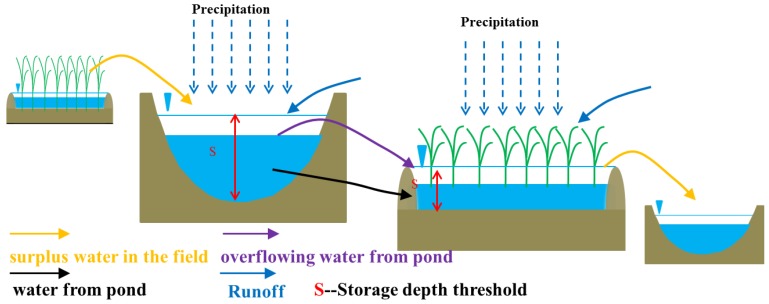
Schematic of water flow between ponds and farmland.

Based on the above principles, the order of priority for both water supply by sources and water intake by users were defined as follows:

The order of priority for water supply by water sources is as follows. Use the water overflow from the upstream pond first, and then the water from the pond in the farmland. On farmland upstream of the pond irrigation system, priority is given to rainwater collection and water overflow from the upstream pond. When upstream farmland water storage reaches the storage depth threshold, the surplus water enters pond storage. After reaching full pond capacity, water overflows out of the pond. Upstream farmland irrigation should use water overflowing from the upstream pond and the surplus water in the field, and any deficit should be supplied by the ponds in the farmland. For farmland downstream of the pond irrigation system, priority is given to the rainwater collection and water overflow from the upstream pond. When farmland water storage reaches the storage depth threshold, the surplus water discharges into the downstream pond system. Downstream farmland irrigation should use water in the pond and the surplus water in the field, and any deficit should be supplied by the ponds. Figure 3 shows a schematic of water flow between ponds and farmland in a simulation unit.

The priority order of water intake by water users is as follows. Meet basic water demand first (some ponds also supply rural domestic water), and then production requirements (i.e., agricultural irrigation). To be specific, priority is given to basic water demand before allocating water for irrigation.

**Figure 4:**
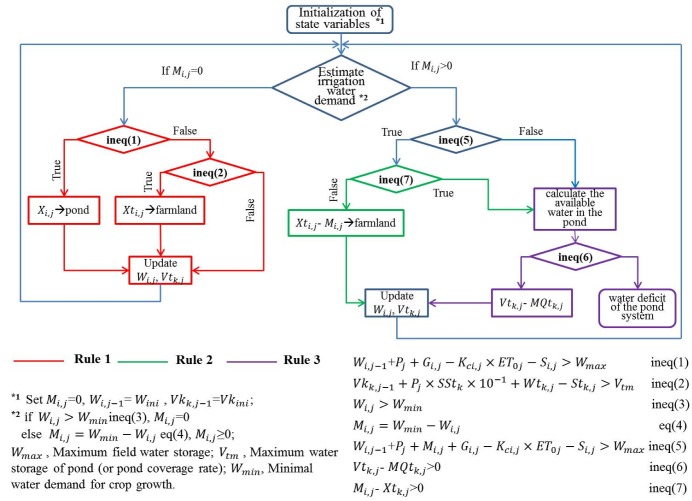
Simulation flow chart of a basic calculation unit.

According to pond irrigation water allocation principles and the order of priority for water supply and water intake, in the simulation model the specific rules for allocating water in the pond irrigation system are as follows (also shown in Figure 4).

First, estimate the irrigation water demand of a crop. If it is zero, follow Rule 1; otherwise, the irrigation water demand is set to the initial water deficit of the system. Next, judge the surplus water in the farmland. If there is surplus water, follow Rule 2; otherwise, follow Rule 3.

Rule 1: If farmland water storage exceeds the storage depth threshold, the surplus water in the farmland flows into the pond; otherwise, the pond inflow (i.e., sum of water overflowing from the upstream pond and the runoff yield in the pond irrigation district) is used to replenish field water storage. The surplus water in the pond control district should be used to recharge the pond, and after reaching full pond capacity the water overflows and discharges outside the pond irrigation district.

Rule 2: If the pond inflow is greater than the initial water deficit in the farmland, follow Rule 3; otherwise, pond inflow should be used to replenish farmland water storage. If farmland water storage reaches the storage depth threshold, the surplus water is used to recharge the pond. After reaching full pond capacity, the water overflows and discharges outside the pond irrigation district.

Rule 3: The available water in the pond is calculated and then subtracted from the water deficit according to Rule 2. If there is still available water, it is the final water deficit of the pond system.

#### 3.2.4. Development of the Water Allocation Simulation Model for the Pond Irrigation System and Parameter Calibration

After developing water allocation rules of the pond irrigation system for the simulation model, various calculation units were connected with their basic parameters according to the direction of water flow in the actual water resources system and supply relationships. The calculation program for the simulation model was written according to the water allocation rules, which can simulate water allocation in the pond irrigation system [15]. Table 1 shows the main steps and Pseudo Code for the irrigation simulation model.

Many model parameters were involved the development of the water allocation simulation model for the pond irrigation system, such as crop irrigation system, the empirical formula or conversion coefficient of water consumption by a crop under deficit irrigation conditions, the effective utilization coefficient of farmland irrigation water, and basic soil parameters (refer to Table S1 in the supplementary file). For the simulation model to be applicable to a particular situation, the values of these parameters have to be calibrated. In this study, using experience and intelligent optimization, the model parameters were calibrated using the following steps.

(1) Several field surveys were conducted. The results were combined with results from crop irrigation experiments conducted by the Water Resources Research Institute of Anhui Province and Huaihe River Commission in the Jiang-Huai hilly regions over the years with technical service experience in agricultural irrigation. At present, the staple crop of Badou Town is rice. Due to the basic properties of rice, conventional broad irrigation is primarily used. For dry crops, supplementary seedling-preservation irrigation is typically used. The conventional irrigation method in this district was quantified as follows. ① For rice, if there is available water in the water source for irrigation, “flood irrigation” is used; if the water depth of the rice field is zero (i.e., *hc* = 0 mm), rice is irrigated until the water depth of the field reaches 50 mm. ② For dry crops, if there is available water in the water source and the dry crops are subject to drought stress (i.e., the average soil moisture at the depth of 0~40 cm is less than 50% of the soil field capacity) for 5 d, supplementary seedling-preservation irrigation is used until the average soil moisture at 0~40 cm depth reaches 90% of soil field water capacity. ③ The storage depth threshold of this district was set to be the same as the upper limit of irrigation water depth, i.e., *Hm* = 50 mm.
(2) The basic parameters of the soil were determined based on the combined experimental results from Badou irrigation experimental station with general soil survey data for Anhui Province. The saturated moisture content of soil at 0–40 cm depth, *θ_b_* = 0.35; field moisture capacity, *θ_t_* = 0.29; wilting moisture content, *θ_t_* = 0.16; soil depth of dry crops for calculation, *H*_1_ = 400 mm; soil depth of rice for calculation, *H*_2_ = 300 mm; and dry density of soil at the calculation soil depth, γ = 1.42 g/cm^3^.
(3) The empirical formulas or conversion coefficients of water consumption for rice and dry crops (i.e., wheat) under deficit irrigation or drought stress conditions were developed according to the crop irrigation and drought experiment results at Badou irrigation experimental station.

Equation (11) shows the empirical formulas for actual transpiration after different water cut-off days for rice. For dry crops, if the soil moisture is 0.5*θ_t_* <*θ* < 0.75*θ_t_*, the average conversion coefficient of crop transpiration *φ*_1_ = 0.8. So, *e_ai_* = 0.8 × *ETc_i_*. If the soil moisture is 0.35*θ_t_* < *θ* < 0.5*θ_t_*, the average conversion coefficient of crop transpiration *φ*_2_ = 0.4. So, *e_ai_* = 0.4 × *ETc_i_*.

(11) $$e_{a}=\{\begin{array}{cc} ET_{c}\left(1.70\times 10^{-5}T^{3}-9.24\times 10^{-4}T^{2}+1.23\times 10^{-3}T+0.998\right) & \;\mathrm{Tillering}\;\mathrm{stage}\\ ET_{c}\left(2.80\times 10^{-5}T^{3}-2.98\times 10^{-4}T^{2}-2.41\times 10^{-2}T+1.005\right) & \;\mathrm{Jointing}\;\mathrm{stage}\\ ET_{c}\left(-2.00\times 10^{-4}T^{3}+5.80\times 10^{-3}T^{2}-7.52\times 10^{-2}T+1.0\right) & \;\mathrm{Heading-to-flowering}\;\mathrm{stage}\\ ET_{c}\left(-1.80\times 10^{-5}T^{3}+1.41\times 10^{-3}T^{2}-4.14\times 10^{-2}T+1.004\right) & \;\mathrm{Maturity}\;\mathrm{stage} \end{array}$$

where, *ETc* is the transpiration (mm) of rice under full irrigation; e_a_ is the actual transpiration (mm) of rice under water deficit; *T* is water cut-off days for rice, where tillering stage *T* < 30 d, jointing stage *T* < 20 d, heading-to-flowering stage *T* < 15 d, and milky maturity stage *T* < 20 d.

### 3.3. Optimal Water Resources Regulation for the Pond Irrigation System

To guarantee the security of regional basic water demand and optimize water resources regulation, regional economic benefits were maximized in this study. Further, an optimization objective function was developed for water allocation simulation. The simulation model parameters were used as optimal regulation parameters to develop the simulation-based optimal water resources regulation model for the pond irrigation system. It also used an accelerating genetic algorithm to solve the optimal regulation model [30,31]. The details are as follows.

#### Development of the simulation-based optimal water resources regulation model for the pond irrigation system

(1) Objective function

Optimal water resources regulation for the pond irrigation system aims to maximize regional economic benefits and guarantee the security of regional basic water demand. Some ponds serve both the functions of water-storage irrigation and rural domestic water supply. The objective function is written as:

(12) $$F=\max\left\{\sum_{i=1}^{m}\sum_{j}^{n}\delta_{ij}B_{ij}Y_{ij}-\sum_{j}^{n}Int_{j}\right\}$$

where, *δ_ij_* is the price (RMB/kg) of the *i^th^* crop in the *j^th^* year; *B_ij_* is the planting area (hm^2^) of the *i^th^* crop in the *j^th^* year,; *Y_ij_* is the yield (kg) of the *i^th^* crop in the *j^th^* year; and *Int_j_* is the average investment (RMB) in the *j^th^* year for pond dredging and capacity expansion projects. In this study, *δ_ij_* was assigned according to the *Statistical Yearbook of Feidong County* for related years. *Y_ij_* was determined from the irrigation water supply for each crop according to the crop water model. *Int_j_* was determined by multiplying the average machinery, manpower, engineering materials and other inputs per unit capacity for pond dredging and expansion excavation by the price indices for related years. The multi-year average benefit of crops and investment of pond expansion used for the calculation can be referred to Table S2 in the supplementary file.

(2) Constraints

(1) The equation for the field water balance constraint is:
  (13) $$W_{i,j}=W_{i,j-1}+P_{j}+M_{i,j}+G_{i,j}-ETc_{i,j}-S_{i,j}-X_{i,j}$$
  (14) $$W_{i,j}=W_{i,j-1}+P_{j}+M_{i,j}+G_{i,j}-ETc_{i,j}-S_{i,j}-X_{i,j}$$
(2) The equation for the pond water balance constraint is:
  (15) $$Vt_{k}{}_{,j}=Vk_{k,j-1}+P_{j}\times SSt_{k}\times 10^{-1}+Wt_{k,j}-MQt_{k,j}-St_{k,j}-Xt_{k,j}$$
(3) Other constraints include the guaranteed irrigation rate constraint (i.e., the simulated guaranteed irrigation rate of the pond irrigation district should be the same as the actual guaranteed irrigation rate for Feidong County); the non-negativity constraint over design parameters (i.e., no simulated pond parameter can be negative); and the maximum water delivery flow restraint over irrigation water supply channels (i.e., the simulated water delivery flow of an irrigation water supply channel cannot exceed its maximum water delivery flow).

## 4. Results and Discussion

### 4.1. Validation of the Pond Irrigation Water Allocation Simulation Model

Using actual daily water consumption for a typical year (2010) as the water demand of the simulation model, the model was optimized by minimizing the square sum of the differences between the simulated and measured pond water intakes for irrigation in the experimental district which was irrigated by four ponds with observed data. The accelerating genetic algorithm [30] was used to optimize and calibrate the effective utilization coefficient of irrigation water and other parameters. The NSE (Nash-Sutcliffe efficiency coefficient) of observed and simulated irrigation water consumption from ponds was 0.78, which can be referred to Figure S1 in the supplementary file. The optimized effective utilization coefficient of irrigation water *α* = 0.664. According to the results from a previous study in Anhui Province from 2007–2012 [28], the effective utilization coefficient of irrigation water for small ponds in the Jiang-Huai hilly regions ranged between 0.653–0.688. Hence, the calibrated effective coefficient agreed with the calculated coefficient.

The simulated pond re-storage times in the Yangdian Town area in 2010 was 1.224. According to measured rainfall and water levels from four ponds in 2009–2011, re-storage times ranged between 1.214 and 1.416. Thus, it is clear that the simulated pond re-storage times obtained by the water allocation simulation model for the pond irrigation system developed in this study not only agreed with measurements, but also with calculated times from previous research [10]. These results show that it is feasible to use the simulation model to simulate the water allocation for the pond irrigation system.

### 4.2. Solving the Simulation-Based Optimal Water Resources Regulation Model for the Pond Irrigation System

#### 4.2.1. Analysis of the Water-Saving Irrigation Method Benefits

Experimental results for Badou and other irrigation experimental stations in the Jiang-Huai hilly regions over the years have shown that intermittent irrigation is one of the most effective water-saving irrigation methods for rice. In addition, the rice field storage depth threshold influences the direct utilization of rainfall by rice. Hence, raising the storage depth threshold by changing rice irrigation and drainage methods can result in high water-saving potential [32]. This study analyzed the suitable intermittent days of rice during different growth stages and the water-saving potential and benefits under the storage depth threshold. The following water-saving irrigation method for this district was used: ① Using the intermittent irrigation method for rice, there was a 30–40 mm water layer in the returning green stage. After the water layer was dry for 7 d in the tillering stage, irrigation occurred until it reached 50 mm. Then, irrigation was repeated until the end of the tillering stage, and field drying occurred for one week in the late tillering stage. After the water layer was dry for 6 d in the jointing stage, irrigation occurred until the water layer was 50 mm. Then, irrigation was repeated until the end of jointing stage. After the water layer was dry for 6 d in the heading-to-flowering stage, irrigation occurred until the water layer was 50 mm. Then, irrigation was repeated until the end of heading-to-flowering stage. After the water layer was dry for 7 d in the maturity stage, irrigation occurred until the water layer was 50 mm. Then, irrigation was repeated until the end of maturity stage. The water layer naturally dried during the final yellow ripening stage. ② For the storage depth threshold of rice field *H_m_*, during the seedling stage, *H_m_* = 50 mm; during the tillering stage, *H_m_* = 100 mm; and during the jointing, heading-to-flowering, and maturity stages, *H_m_* = 150 mm.

**Figure 5:**
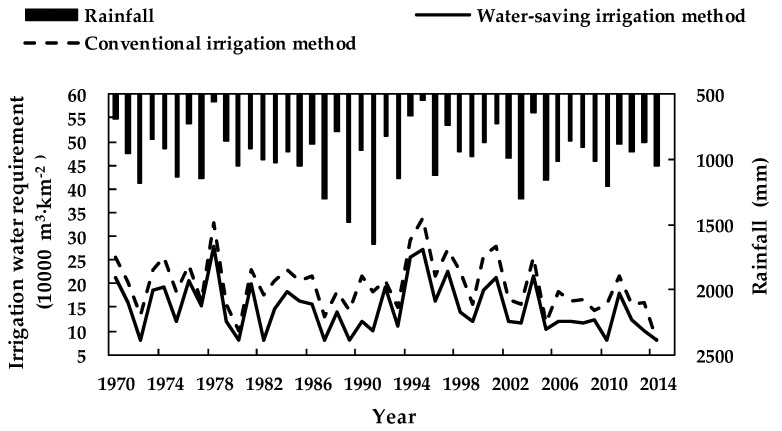
Annual irrigation crop water requirements under conventional and water-saving irrigation methods in the pond irrigation district of Yangdain Town from 1970–2014.

To quantify the water-saving potential of the selected water-saving irrigation method, the irrigation water requirements, water deficits, and crop revenues of the crops under conventional and water-saving irrigation methods were simulated [33]. A crop’s irrigation water requirement is its water consumption under an irrigation method when there is sufficient available water at the supply source (i.e., the maximum irrigation water consumption of the crop, which can be calculated using Equation (5)). The water deficit is the difference between the crop irrigation water requirement and pond water supply under an irrigation method. Crop revenue is the product of crop yield and price. Figure 5 and Table 2 show the results of the simulation.

From Figure 5 and Table 2, it can be seen that under the current crop planting structure conditions in the pond irrigation district of Yangdian Town, the water-saving irrigation method developed in this study reduced both crop irrigation water requirements and the water deficit. Further, it improved crop irrigation water supply and demand compared to the conventional irrigation method. The details are as follows: (1) The multi-year average irrigation water requirement decreased from 1.97 × 10^5^ m^3^/km^2^ to 1.49 × 10^5^ m^3^/km^2^; the multi-year average water deficit decreased from 48,300 m^3^/km^2^ to 27,700 m^3^/km^2^; and the multi-year average water deficit ratio decreased from 20.40% to 14.06%. These results show that the water-saving irrigation method significantly reduced crop water requirements along with the difference between crop irrigation supply and demand. (2) Multi-year average crop revenue increased by 5.53 × 10^4^ RMB (8035$)/km^2^ and multi-year average water-saving revenue increased by 3.34%. To be specific, in the dry years (75% ≤ *P* < 90%), annual average crop revenue increased by 1.18 × 10^5^ RMB (17,145$)/km^2^ and water-saving revenue increased by 7.86%. In drought years (*P* ≥ 90%), annual average crop revenue increased by 1.49 × 10^5^ RMB (21,649$)/km^2^ and water-saving revenue increased by 11.10%. (3) For rainfall frequency *P* ≥ 75%, the crop irrigation water deficit ratio exceeded 29.85% even when the water-saving irrigation method developed in this study was used. Therefore, serious drought-incurred losses are unavoidable. The current crop planting structure was unsustainable and the current pond coverage rate of 1.03 × 10^5^ m^3^/km^2^ was a little low. In order to apply the water-saving irrigation method, it was first necessary to determine the optimal pond coverage rate of the study area and adjust the crop planting structure.

#### 4.2.2. Simulation-Based Analysis of Water Resources Regulation for the Pond Irrigation System

After using parameters from the water-saving irrigation method as inputs to the simulation model, the pond coverage rate and rice planting proportions under different rainfall frequencies and different initial annual pond water storage values were introduced as regulatory variables. Using Equation (12) as the objective function, the accelerating genetic algorithm [31] solved the water resources regulation model (Section 4.1) for the pond coverage rate *V_tm_* = 2.92 × 10^5^ m^3^/km^2^. The suitable rice planting proportions under different rainfall frequencies (*P*) and different initial annual pond initial water storages were as follows.

(1) For *P* < 20% (wet year), ① if the initial water storage *Vts* > 2 × 10^5^ m^3^/km^2^, the suitable planting proportion of autumn rice was 0.676. ② If the initial water storage 10 < *Vts* ≤ 2 × 10^5^ m^3^/km^2^, it was 0.653. ③ If the initial water storage *Vts* ≤ 1 × 10^5^ m^3^/km^2^, it was 0.632.
(2) For 20% ≤ *P* < 50% (relatively wet year), ① if the initial water storage *Vts* > 2 × 10^5^ m^3^/km^2^, the suitable planting proportion of autumn rice was 0.652. ② If the initial water storage 10 < Vts ≤ 2 × 10^5^ m^3^/km^2^, it was 0.622. ③ If the initial water storage *Vts* ≤ 100,000 m^3^/km^2^, it was 0.592.
(3) For 50% ≤ *P* < 75% (relatively dry year), ① if the initial water storage *Vts* > 2 × 10^5^ m^3^/km^2^, the suitable planting proportion of autumn rice was 0.648. ② If the initial water storage 10 < *Vts* ≤ 2 × 10^5^ m^3^/km^2^, it was 0.504. ③ If the initial water storage *Vts* ≤ 100,000 m^3^/km^2^, it was 0.422.
(4) For 75% ≤ *P* < 90% (dry year), ① if the initial water storage *Vts* > 2 × 10^5^ m^3^/km^2^, the suitable planting proportion of autumn rice was 0.598. ② If the initial water storage 10 < *Vts* ≤ 2 × 10^5^ m^3^/km^2^, it was 0.456. ③ If the initial water storage *Vts* ≤ 1 × 10^5^ m^3^/km^2^, it was 0.324.
(5) For *P* ≥ 90% (drought year): ① if the initial water storage *Vts* > 2 × 10^5^ m^3^/km^2^, the suitable planting proportion of autumn rice was 0.458. ② If the initial water storage 10 < *Vts* ≤ 2 × 10^5^ m^3^/km^2^, it was 0.302. ③ If the initial water storage *Vts* ≤ 1 × 10^5^ m^3^/km^2^, it was 0.223.

An optimal water resources regulation model for the pond irrigation system was developed based on the above results. The details are as follows: ① During pond dredging and expansion excavation, the pond coverage rate increased from 1.03 × 10^5^ m^3^/km^2^ to *V_tm_*= 2.92 × 10^5^ m^3^/km^2^. ② The crop planting structure was optimized. The details were in the suitable rice planting proportions under different rainfall frequencies and different initial annual pond water storages. ③ The intermittent irrigation method was used for rice, with a 30–40 mm water layer in the returning green stage. After the water layer was dry for 7d in the tillering stage, irrigation was provided until there is a water layer of 50 mm. Then, the irrigation was repeated until the end of tillering stage, and field drying occurred for one week in the late tillering stage. After the water layer was dry for 6d in the jointing stage, irrigation occurred until there is a water layer of 50 mm. Then, irrigation was repeated until the end of jointing stage. After the water layer was dry for 6d in the heading-to-flowering stage, irrigation occurred until there is a water layer of 50 mm. Then, irrigation was repeated until the end of heading-to-flowering stage. After the water layer was dry for 7d in the maturity stage, irrigation occurred until there was a water layer of 50 mm. Then, irrigation was repeated until the end of maturity stage, and it naturally dried in the final yellow ripening stage. ④ The storage depth threshold of rice field increased. For the storage depth threshold of rice field *H_m_*, during the seedling stage, *H_m_*= 50 mm; during the tillering stage, *H_m_*= 100 mm; and during the jointing, heading-to-flowering, and maturity stages, *H_m_*= 150 mm. After substituting the related parameters for the optimal regulation mode into the series water resources regulation model for the pond irrigation system, the allocation process and crop revenue of annual irrigation water supply and demand for the pond irrigation system were simulated. Average crop revenues under different rainfall frequencies were analyzed and the results were compared with the conventional irrigation method. Calculation results are shown in Table 3.

**Figure 6:**
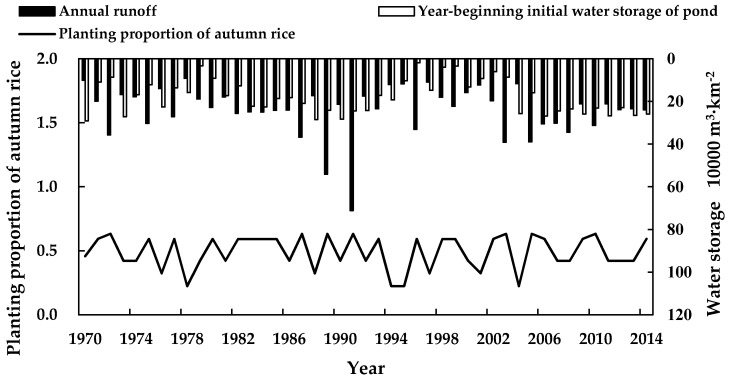
Annual suitable planting proportions for autumn rice under the optimal regulation mode for the pond irrigation district of Yangdain Town from 1970–2014.

From Figure 6 and Table 3, it can be seen that under the optimal water resources regulation mode, crop irrigation water supply and demand were improved, and the water-saving benefit increased significantly. The details are as follows.

(1) Increasing the planting proportion of autumn rice in the study area caused the irrigation water requirement of the crop to be significantly lower. Further, it reduced the multi-year average total irrigation water requirement by 6.04 × 10^4^ m^3^/km^2^, or 30.6%. These results show that the optimal regulation mode developed in this study reduced the difference between the supply and demand in the irrigation district from the demand side, resulting in water-saving.
(2) The optimal suitable pond coverage rate of the study area was 2.92 × 10^5^ m^3^/km^2^, which was consistent with local multi-year average runoff. Water regulation and storage effectively increased the interception, storage, and utilization of runoff, and reduced the multi-year average surplus water of the pond irrigation system by 3.95 × 10^4^ m^3^/km^2^, or 56.2%.
(3) Optimizing the crop planting structure improved the planting proportion of autumn rice (i.e., the multi-year average planting proportion of autumn rice increased from 0.48 to 0.52); reduced both the water deficit and water deficit ratio (i.e., the multi-year average total irrigation water deficit decreased by 4.66 × 10^4^ m^3^/km^2^, and the multi-year average water deficit ratio decreased from 20.40% to 1.18%); and significantly increased crop revenues (i.e., the multi-year average crop revenue increased by 1.11 × 10^5^ RMB (16,128$)/km^2^, and multi-year average revenue increased by 6.69%). To be specific, in wet years (*P* < 20%), annual average crop revenues increased by 5.72 × 10^4^ RMB (8311$)/km^2^ at a rate of 3.25%. In relatively wet years (20% ≤ *P* < 50%), annual average crop revenues increased by 7.04 × 10^4^ RMB (10229$)/km^2^, at a rate of 4.08%. In relatively dry years (50% ≤ *P* < 75%), annual average crop revenues increased by 6.81 × 10^4^ RMB (9895$)/km^2^, at a rate of 4.06%. In dry years (75% ≤ *P* < 90%), annual average crop revenue increased by 2.04 × 10^5^ RMB (29641$)/km^2^, at a rate of 13.65%. In drought years (*P* ≥ 90), annual average crop revenues increased by 3.50 × 10^5^ RMB (50,854$)/km^2^, at a rate of 26.09%.

#### 4.2.3. Implication of Optimal Water Resources Regulation on the Environments

Because irrigation systems deal with redirecting water from rivers, lakes, and underground water, they have a direct impact on the surrounding environment. Some of these impacts include: increased groundwater level in irrigated areas, waterlogging, soil salinization and increased evaporation in irrigated areas [34]. On the premise of plant normal growth, the optimal water resources regulation measures based on modeling in this study can reduce the “flooding irrigation” to the most extent, and then, reduce the risk of waterlogging and soil salinization and unnecessary evaporation loss. Water saved by the optimal regulation in the ponds can be used for other ecological environment function, such as recharging sourced rivers and streams.

## 5. Conclusions

Pond irrigation has a long history in the Jiang-Huai hilly regions and plays an essential role in agriculture. This study used the system simulation principle and an intelligent optimization algorithm. Further, a simulation-based optimal water resources regulation technology system was developed for the pond irrigation system. Based on this system, the suitable pond coverage rate was determined to be 2.92 × 10^5^ m^3^/km^2^. The suitable irrigation method and storage depth threshold for rice, along with water supply rules for the pond irrigation district were calculated. Rules for adjusting the crop planting structure were developed based on the annual rainfall depth and the initial annual pond water storage values. By comparing water supply and demand, and crop revenue, with the conventional irrigation method, the rationality and effectiveness of the model system was demonstrated. To guarantee rural domestic water demand, multi-year average total irrigation water deficit decreased by 4.66 × 10^4^ m^3^/km^2^; the multi-year average water deficit ratio decreased from 20.40% to 1.18%; the multi-year average crop revenue increased by 1.11 × 10^5^ RMB (16128$)/km^2^; and the multi-year average revenue increased by 6.69%. Model results also increased runoff interception, storage, and utilization rates in the pond irrigation district, thereby improving the annual pond water supply capacity. This significantly improved their drought resistance and disaster reduction capacity, with associated economic, social and environmental benefits. The results of this study can provide the theoretical basis and technological support for comprehensive pond governance in the Jiang-Huai hilly regions, and inform the establishment of a water allocation scheme and irrigation system for pond irrigation districts, which have important application value.

## Figures and Tables

**Table 1:** Main steps and Pseudo Code for the irrigation simulation model.

Steps	Pseudo-Code of the Model Calculation Process
1	load the model inputs, which includes Daily Precipitation, Surface Runoff (calculated from SCS model) and Potential Evapotranspiration (calculated from PM formula)
2	Decide the maximal day without irrigation for different crop growth periods
3	Decide rainfall storage depth for different growth periods for the rice field
4	Calculate the daily actual evapotranspiration of crops
5	Field water balance calculation: Initialize field water depth (for rice), field water storage, initial soil moisture content, set maximum field water storage for different crop growth periods Loop: (1) estimate the irrigation water demand; (2) calculate the surplus water in the farmland; (3) update field water depth, field water storage and soil moisture content;
6	Calculate the irrigation water consumption needed from pond by the result of step 5 and the effective utilization coefficient of farmland irrigation water
7	Pond water balance calculation: Initialize the water storage of the pond Loop: (1) calculate the actual irrigation water supply from the pond; (2) calculate the surplus water of the pond and water deficit; (3) update water storage of the pond;
8	Calculate the average crop revenues, irrigation water requirement, pond water supply, water deficit and other objective function

**Table 2:** Crop water deficits and deficit ratios under conventional and water-saving irrigation methods in the pond irrigation district of Yangdian Town.

Rainfall Frequency	Operation Mode	Irrigation Water Requirement (unit:*^1^)	Pond Water Supply (unit:*^1^)	Water Deficit (unit:*^1^)	Water Deficit Ratio (%)	Crop Revenue (unit:*^2^)
*P* < 20%	Conventional irrigation method	14.50	13.71	0.79	6.67	176.07
	Water-saving irrigation method	9.24	8.58	0.66	6.42	176.37
	Water-saving effect	−5.26	−5.13	−0.13	−0.26	0.30
20% ≤ *P* < 50%	Conventional irrigation method	17.42	15.10	2.32	12.43	172.48
	Water-saving irrigation method	12.93	12.14	0.79	5.96	176.05
	Water-saving effect	−4.48	−2.95	−1.53	−6.47	3.57
50% ≤ *P* < 75%	Conventional irrigation method	20.28	16.04	4.23	19.61	167.70
	Water-saving irrigation method	15.36	13.16	2.19	12.59	172.93
	Water-saving effect	−4.92	−2.88	−2.04	−7.02	5.23
75% ≤ *P* < 90%	Conventional irrigation method	24.38	14.18	10.20	38.79	149.59
	Water-saving irrigation method	19.60	13.16	6.44	29.85	161.35
	Water-saving effect	−4.79	−1.02	−3.77	−8.94	11.76
*P* ≥ 90%	Conventional irrigation method	29.39	68.01	15.79	52.48	134.22
	Water-saving irrigation method	24.59	13.98	10.61	41.86	149.12
	Water-saving effect	−4.80	−54.03	−5.18	−10.62	14.90
Multi-year average	Conventional irrigation method	19.74	14.91	4.83	20.40	165.37
	Water-saving irrigation method	14.95	12.18	2.77	14.06	170.90
	Water-saving effect	−4.79	−2.73	−2.06	−6.34	5.53

Note: *1: 1 × 10^4^ m^3^/km^2^; *2: 1 × 10^4^ RMB (1482$)/km^2^.

**Table 3:** Comparison of average crop revenues between the conventional method and optimal regulation mode under different rainfall frequencies in the pond irrigation district of Yangdian Town.

Rainfall Frequency	Operation Mode	Irrigation Water Requirement (unit:*^1^)	Surplus Water (unit:*^1^)	Water Deficit (unit:*^1^)	Water Deficit Ratio (%)	Crop Revenue (unit: *^2^)
*P* < 20%	Conventional irrigation method	14.50	22.46	0.79	6.67	176.07
	Optimal regulation mode	11.23	13.90	0.00	0.00	181.79
	Optimal regulation effect	−3.27	−8.56	−0.79	−6.67	5.72
20% ≤ *P* < 50%	Conventional irrigation method	17.42	4.94	2.32	12.43	172.48
	Optimal regulation mode	14.60	0.51	0.38	2.53	179.52
	Optimal regulation effect	-2.81	-4.43	-1.94	-9.90	7.04
50% ≤ *P* < 75%	Conventional irrigation method	20.28	5.15	4.23	19.61	167.70
	Optimal regulation mode	12.96	2.40	0.00	0.00	174.51
	Optimal regulation effect	-7.32	-2.75	-4.23	-19.61	6.81
75% ≤ *P* < 90%	Conventional irrigation method	24.38	1.84	10.20	38.79	149.59
	Optimal regulation mode	14.05	0.35	0.47	3.15	170.01
	Optimal regulation effect	−10.33	−1.49	−9.73	−35.64	20.42
*P* ≥ 90%	Conventional irrigation method	29.39	1.14	15.79	52.48	134.22
	Optimal regulation mode	15.94	0.07	0.00	0.00	169.24
	Optimal regulation effect	−13.45	−1.07	−15.79	−52.48	35.02
Multi-year average	Conventional irrigation method	19.74	7.03	4.83	20.40	165.37
	Optimal regulation mode	13.70	3.08	0.18	1.18	176.44
	Optimal regulation effect	−6.04	−3.95	−4.66	−19.22	11.07

Note: *1: 1 × 10^4^ m^3^/km^2^; *2: 1 × 10^4^ RMB (1482$)/km^2^.

## Supplementary Materials

The following are available online at https://www.mdpi.com/1660-4601/16/15/2717/s1.
