# Supplementary material for: Optimal Water Resources Regulation for the Pond Irrigation System Based on Simulation—A Case Study in Jiang-Huai Hilly Regions, China
Source: Int J Environ Res Public Health. 2019 Jul 30;16(15):2717. doi: 10.3390/ijerph16152717 (PMC6695888; doi:10.3390/ijerph16152717)
Supplement: Supplementary file 1 [file ijerph-16-02717-s001.pdf]

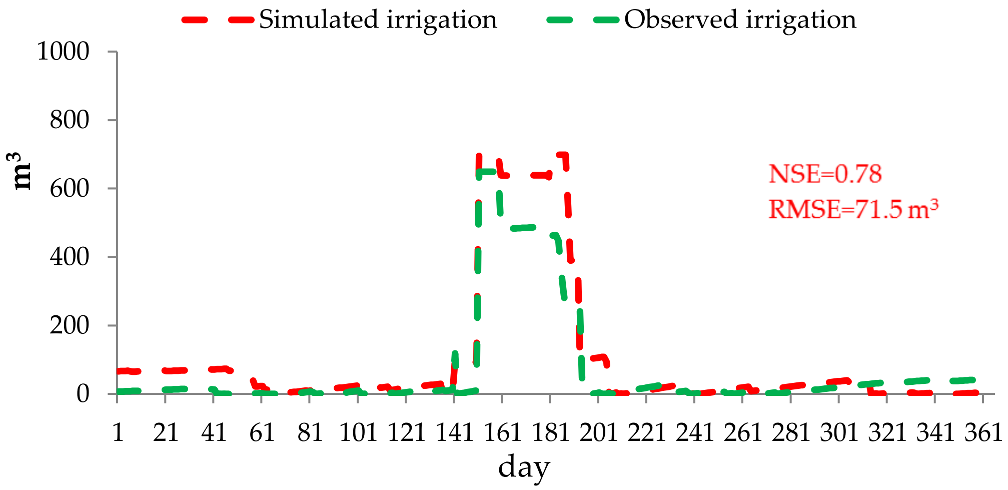

Figure S1. Observed and simulated irrigation water consumption from ponds.

Figure S1 shows the observed and simulated irrigation water consumption in the experimental district which was irrigated by four ponds. Because most of the study area is ungauged, we only collect one year data for model calibration, and also our model is like a water resource allocation model which is not as complicated as other hydrological models that need long warming up period. Besides that, we used effective utilization coefficient of irrigation water and pond re-storage times in the Badou Town for validation. These results show that it is feasible to use the simulation model to simulate the water allocation for the pond irrigation system.

Table S1. The parameters used in the irrigation simulation model

Parameter Name	Description	Range	Value	Set by
Kc_{11}	The coefficient of the actual evapotranspiration for rice in the tillering stage	0～2	1.133	automatic calibration
Kc_{12}	The coefficient of the actual evapotranspiration for rice in the joining stage	0～2	1.251	automatic calibration
Kc_{13}	The coefficient of the actual evapotranspiration for rice in the heading-to-flowering stage	0～2	1.445	automatic calibration
Kc_{14}	The coefficient of the actual evapotranspiration for rice in the maturity stage	0～2	1.237	automatic calibration
Kc_{21}	The coefficient of the actual evapotranspiration for wheat in the tillering stage	0～2	0.98	automatic calibration
Kc_{22}	The coefficient of the actual evapotranspiration for wheat in the joining stage	0～2	1.228	automatic calibration
Kc_{23}	The coefficient of the actual evapotranspiration for wheat in the heading-to-flowering stage	0～2	1.35	automatic calibration
Kc_{24}	The coefficient of the actual evapotranspiration for wheat in the maturity stage	0～2	1.05	automatic calibration
Kc_{31}	The coefficient of the actual evapotranspiration for corn in the tillering stage	0～2	0.985	automatic calibration
Kc_{32}	The coefficient of the actual evapotranspiration for corn in the joining stage	0～2	1.124	automatic calibration
Kc_{33}	The coefficient of the actual evapotranspiration for corn in the heading-to-flowering stage	0～2	1.217	automatic calibration
Kc_{34}	The coefficient of the actual evapotranspiration for corn in the maturity stage	0～2	1.073	automatic calibration
Zh	Reduction factor of transpiration ,(water requirement) for crop under drought condition	0～1	0.1	automatic calibration
Zf	Reduction factor of transpiration ,(water requirement) for crop under non-full irrigation	0～1	0.6	automatic calibration

Kt	The coefficient of evaporation capacity of water surface for the pond	>1	1.1	automatic calibration
α	Effective utilization coefficient of farmland irrigation water	0～1	0.664	automatic calibration
hc	The water or irrigation depth of the field (mm) (for dry crops, *hc* = 0)	0～200	50mm	experiment or long-term observation
H_1	Soil depth of dry crops for calculation	>0	400mm	experiment or long-term observation
H_2	Soil depth of rice for calculation	>0	300mm	experiment or long-term observation
γ	The dry density of soil (g/cm^3) at the calculated soil depth	>0	1.42 g/cm^3	experiment or long-term observation
θ_b	The saturated moisture content of soil at 0~40 cm depth	0～1	0.35	experiment or long-term observation
θ_f	Field moisture capacity	0～1	0.29	experiment or long-term observation
θ_t	Wilting moisture content	0～1	0.16	experiment or long-term observation
Hm	The storage depth threshold	>0	50mm	experiment or long-term observation

Table S2. The multi-year average benefit of crops and investment of pond expansion

Item	Benefits and Expenses
Rice (Unit [1])	132.01
Corn (Unit [1])	60.00
Wheat (Unit [1])	75.52
Average investment for pond dredging and capacity expansion (unit [2])	105.13

Note: Unit[1]: 1×10^4 RMB (1482$)/km^2, Unit[2]: 1×10^4 RMB (1482$)/year

About the parameters used in the SCS model

The SCS model used in this study is the same as model used in the reference 10 (*Transactions of the Chinese Society of Agricultural Engineering*. 2013, 29(18), 117-124; In Chinese; cited by EI), so most of detail was not shown in the main body. The relative details were translated into English and shown as follows:

Using the 5-day rainfall depth preceding the current rainfall, the SCS model classifies the *AMC* into three grades, i.e. *AMCI* for dry, *AMCII* for moderately moist, and *AMCШ* for moist in the study area as Table S3 shows.

Table S3. Antecedent soil moisture level grading standard of study area (Table 3 of reference 10)

Antecedent Moisture Conditions	Antecedent 5-day rainfall (mm)	
	Dormant season	Growing season
AMC Ⅰ	<10	<30
AMC II	10~25	30~50
AMCIII	>25	>50

Based on the proportion of land use class of different towns, feature of different soil types and antecedent Moisture Conditions in the study area, the value of CN_2 can be set by looking up the CN table provided by Soil Conservation Service of United States, then CN_1 and CN_3 can be calculated by equation (3) and (4) (as shown in main body). All the CN values in different towns are shown as follows in Table S4:

Table S4. The different *CN* values of each town in the study area. (Table 4 of reference 10)

CN	Yangdian	**Badou**	Chenji

CN_1	67.6	67.0	67.8
CN_2	83.4	83.0	83.5
CN_3	93.3	93.0	93.3

Daily rainfall at Feidong Station (Figure 1) was used as the input to the SCS model, and the daily runoff was simulated by SCS model with the previously determined parameters. The monthly runoff of different towns was shown in Table S5. The simulated runoff was close to the experimental and observed results of local hydrological test station and engineering practice.

Table S5. The different typical year monthly runoff simulation results of each town in the study area (Units: mm Table 5 of reference 10).

Month	Yangdian			**Badou**			Chenji		
	P=50 %	P=75 %	P=95 %	P=50 %	P=75 %	P=95 %	P=50 %	P=75 %	P=95 %
1	0.5	3.4	4.0	0.4	3.3	5.4	0.5	3.4	4.0
2	0.8	1.0	1.9	0.8	1.0	1.9	0.8	1.0	2.0
3	4.5	8.7	7.2	4.4	8.5	9.6	4.5	8.7	7.3
4	12.4	20.2	14.0	12.4	21.8	14.6	12.4	20.3	14.0
5	8.9	7.8	11.4	11.3	9.6	11.1	8.9	7.8	11.4
6	119.3	47.2	28.8	117.6	46.3	28.2	119.5	47.3	28.9
7	4.3	48.2	10.3	4.2	55.5	10.0	4.3	48.4	10.3
8	52.5	7.3	0.0	51.6	8.7	0.0	52.6	7.3	0.0
9	5.7	18.9	1.4	5.5	18.4	1.4	5.7	19.0	1.4
10	35.2	0.0	3.4	34.4	0.0	3.3	35.3	0.0	3.4
11	2.1	1.2	23.5	2.0	1.1	23.0	2.1	1.2	23.5
12	0.0	3.9	3.8	0.0	4.3	4.3	0.0	3.9	3.8
Total	246.1	167.7	109.7	244.6	178.6	112.7	246.7	168.3	110.1
